# Plausible Protective Role of *Encephalartos villosus* Extract in Acetic-Acid-Induced Ulcerative Colitis in Rats

**DOI:** 10.3390/ph16101431

**Published:** 2023-10-09

**Authors:** Ashwag S. Alanazi, Mohammed M. Alanazi, Engy Elekhnawy, Nashwah G. M. Attallah, Walaa A. Negm, Aya H. El-Kadem

**Affiliations:** 1 Department of Pharmaceutical Sciences, College of Pharmacy, Princess Nourah Bint Abdulrahman University, P.O. Box 84428, Riyadh 11671, Saudi Arabia; asalanzi@pnu.edu.sa; 2 Department of Pharmaceutical Chemistry, College of Pharmacy, King Saud University, P.O. Box 2457, Riyadh 11451, Saudi Arabia; 3 Pharmaceutical Microbiology Department, Faculty of Pharmacy, Tanta University, Tanta 31527, Egypt; 4 The Egyptian Drug Authority (EDA), Previously NODCAR, Giza 8655, Egypt; nashwahattallah@gmail.com; 5 Department of Pharmacognosy, Faculty of Pharmacy, Tanta University, Tanta 31527, Egypt; walaa.negm@pharm.tanta.edu.eg; 6 Department of Pharmacology and Toxicology, Faculty of Pharmacy, Tanta University, Tanta 31527, Egypt

**Keywords:** TLR-4, NF-ĸB, TNF-α, CAT, mucosal integrity

## Abstract

Ulcerative colitis (UC) is an inflammatory ailment of the intestine associated with the upregulation of oxidative stress and pro-inflammatory cytokines. Here, we aimed to assess the consequences of *Encephalartos villosus* (EV) Lem extract on acetic acid (AA)-induced UC. Rats were randomly classified into five groups, as follows: control, AA, AA + mesalazine, AA + EV (50 mg/kg), and AA + EV (100 mg/kg) groups. EV (50 mg/kg and 100 mg/kg) and mesalzine (100 mg/kg) were administered orally for 14 days before the induction of UC. On the last day of the experiment, colitis was provoked via the intra-rectal delivery of 3% AA. Then, after 24 h, the rats were sacrificed and their colon tissues were isolated and inspected. Interestingly, EV pretreatment substantially (*p* < 0.05) reduced the elevated colon weight/length ratio and ulcer area and normalized the histological changes and immunohistochemical features. In addition, EV efficiently reduced the levels of myeloperoxidase (MPO) and increased the activity of glutathione peroxidase (GS-PX) and catalase (CAT). EV (100 mg/kg) resulted in a downregulation of toll-like receptor 4 (TLR-4) and upregulation of heme oxygenase 1 (HO-1) and occludin expression levels. Concerning the anti-inflammatory mechanisms, EV reduced the levels of tumor necrosis factor-alpha (TNF-α), interleukin-6 (IL-6), and nuclear transcription factor kappa B (NF-ĸB) and inhibited cyclooxygenase-2 (COX-2) expression levels. It also decreased caspase-3 levels. Our results indicate that the oral intake of EV improves AA-induced colitis in rats through its antioxidative effects and the modulation of pro-inflammatory cytokines, as well as the restoration of mucosal integrity. Consequently, EV may be an efficient therapeutic candidate for UC.

## 1. Introduction

Ulcerative colitis (UC) and Crohn’s disease (CD) are ailments of the gastrointestinal tract (GIT). Repeated and non-specific inflammation of the intestine usually occurs in these diseases [1,2]. It is hypothesized that the interaction between the environment and genes triggers an immunological response in the mucosa of the intestine, provoking remarkable inflammation, as well as injury, in the GIT [3].

Toll-like receptors (TLRs) are components of the immune system that play an important role in intestinal inflammation [4,5]. There are many toll-like receptor subtypes, and TLR-4 is one of these subtypes, which plays a major role in inflammatory bowel diseases (IBD). In these conditions, the level of TLR-4 increases [4]. As a result of this increase, upregulation typically occurs in the downstream signaling pathways, including the nuclear factor binding kappa light-chain (NF-ĸB) signaling pathway [6]. As a result, this prompts the release of different pro-inflammatory cytokines, like tumor necrosis factor-alpha (TNF-α) [7].

NF-kB has an important role in the pathogenesis of UC by triggering the release of inflammatory mediators, like cyclooxygenase-2 (COX-2) [8,9]. COX-2 then induces the release of prostaglandins through the metabolism of arachidonic acid [10].

The activation of inducible nitric oxide synthase (iNOS), as well as COX-2, usually results in the destruction of intestinal mucosa by inducing the production of free radicals and inhibiting the anti-oxidative system [11,12]. Indeed, the excessive production of reactive oxygen species (ROS) negatively affects the defense system of the mucosa [13].

The increased release of pro-inflammatory mediators, like TNF-α, can trigger apoptosis, which has an important role in animal colitis models. It is reported that apoptosis is associated with the pathophysiology of IBD [14,15]. The inflammatory response changes the function of the mucosal barrier and affects intestinal integrity, leading to the induction of apoptosis [16]. Consequently, finding novel alternatives that inhibit the inflammatory cascade is a favorable approach to improving the pathological consequences of IBD, which is an inflammatory-mediated ailment [17].

*Encephalartos villosus* Lem. is a decorative dwarf cycad often referred to as the poor man’s cycad. A previous phytochemical study on *E. villosus* leaves documented the isolation of four flavone glycosides: luteolin-7-glucoside, luteolin-7-rutinoside, apigenin-7-glucoside, and luteolin-7-rhamnoside. This plant exhibited promising anti-inflammatory effects [18]. Therefore, we aimed to elucidate the anti-inflammatory potential of EV in acetic-acid-induced colitis in rats and to explore the potential underlying mechanisms of such consequences.

## 2. Results

### 2.1. HPLC Analysis

**Figure 1:**
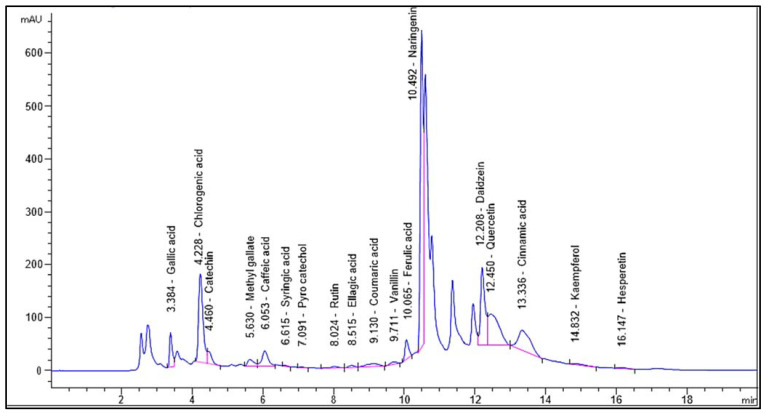
Chromatogram for the identified flavonoids and phenolic compounds of *Encephalartos villosus*.

Identification and quantification of the chemical composition of *Encephalartos villosus* extract was performed using HPLC against standard compounds (Figure S1). Figure 1 shows the HPLC chromatogram for the identified flavonoids and phenolic compounds in the *Encephalartos villosus* extract. The HPLC analysis identified 18 compounds (Table 1). The most abundant phenolic compound was chlorogenic acid (7595.27 μg/g), and the major flavonoid was naringenin (11,476.89 μg/g).

### 2.2. Colon Weight

AA group displayed a noteworthy increase in colon weight (24.82%) compared to the control group. Mesalazine and EV 100 displayed a pronounced reduction (*p* < 0.05) in the colon weight (24.96 and 20.87%, respectively) compared to the AA group. The effect was non-significant in the EV 50 group (Table 2).

### 2.3. Colon Length

The acetic acid (AA) group displayed a noteworthy reduction in colon length (21.7%) compared to the control group. Mesalazine and EV 100 presented a substantial rise (*p* < 0.05) in colon length (18.81 and 23.76%, respectively) compared to the AA group. EV 50 presented no substantial change (4.95%) compared to the AA group (Table 2).

### 2.4. Colon Weight/Length Ratio

Table 2 reveals that the AA group had a substantial rise in the colon weight/length ratio (60.68%) compared to the control group. Mesalazine, EV 50, and EV 100 showed a substantial decline (*p* < 0.05) in the colon weight/length ratio (37.12, 17.89, and 36.5%, respectively) when compared to the AA group, with a more pronounced effect in the EV 100 group compared to the EV 50 group (Table 2).

### 2.5. Colon Catalase (CAT) Activity

**Figure 2:**
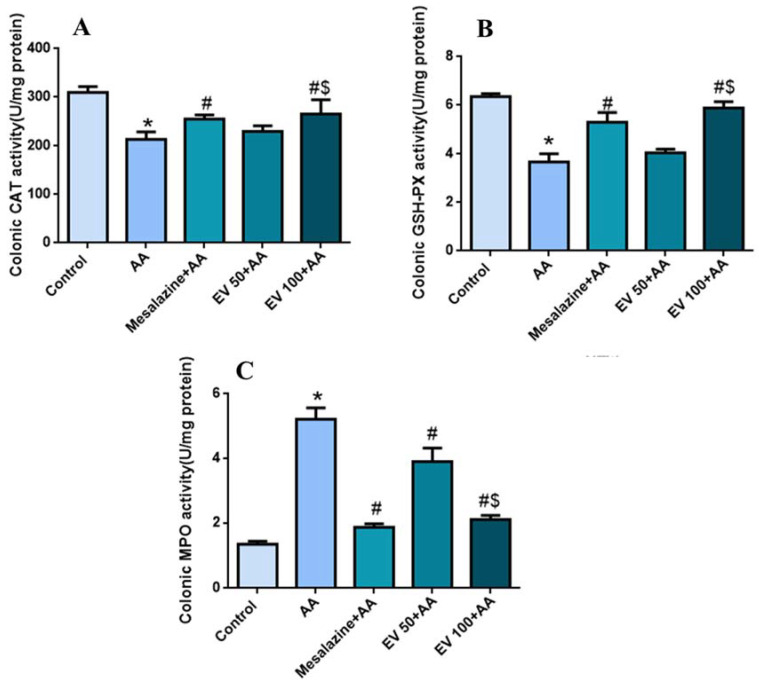
Influence of EV extract on colonic (**A**) CAT (**B**) GSH-PX (**C**) MPO activity in AA-induced UC. Significant difference vs. * respective control, ^#^ respective AA group, and ^$^ respective EV 50 group. *p* < 0.05.

The AA group displayed a significant decrease (*p* < 0.05) in colonic CAT activity (30.93%) compared to the control group. Treatment with mesalazine and EV 100 successfully restored CAT activity (19.7, 7.76, and 24.65%, respectively) compared to the AA group. Moreover, EV 100 showed a more substantial increase (*p* < 0.05) in CAT activity compared to the AA group (24.65%) (Figure 2A).

### 2.6. Colon Glutathione Peroxidase (GSH-PX) Activity

Figure 2B shows a marked reduction in colonic GSH-PX activity in the AA group (42.2%) compared to the control group. The mesalazine-treated group effectively reestablished GSH-PX activity (44.68%) compared to the AA group. EV 50 and EV 100 also induced a substantial increase (*p* < 0.05) in GSH-PX activity compared to the AA group (10.35 and 60.21%, respectively) (Figure 2B); *p* < 0.05.

### 2.7. Colon Myeloperoxidase (MPO) Activity

As shown in Figure 2C, the AA group showed a substantial escalation (*p* < 0.05) in MPO activity (289.55%) compared to the control group. Mesalazine, EV 50, and EV 100 showed a substantial decline (*p* < 0.05) in MPO activity (64.17, 25.28, and 59.57%, respectively) compared to the AA group. MPO activity in the EV 100 group was significantly reduced (88.10%) (Figure 2C).

### 2.8. Colon Gene Expression of TLR-4, Hemoxygenase (HO-1), and Occludin

**Figure 3:**
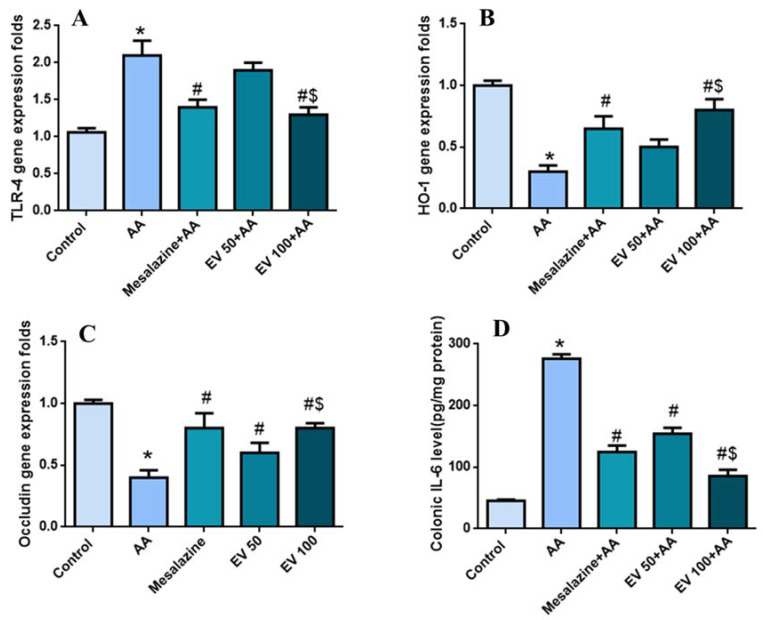
Influence of EV extract on colonic (**A**) TLR-4 gene expression, (**B**) HO-1 gene expression, (**C**) occludin expression, and (**D**) IL-6 level in AA-induced UC. Significant difference vs. * respective control, ^#^ respective AA group, and ^$^ respective EV 50 group; *p* < 0.05.

The AA group displayed a noteworthy rise (*p* < 0.05) in colonic TLR-4 gene expressions (110%) compared to the control group. Mesalazine, EV 50, and EV 100 induced a noteworthy decline (*p* < 0.05) in TLR-4 expression (33.33, 9.5, and 38%, respectively) compared to the AA group. There was no substantial alteration in the TLR-4 expression level in the EV 50 group (6.52%) compared to the AA group (Figure 3A).

Also, in Figure 3B, the AA group revealed a noteworthy decline in colonic HO-1 gene expressions (233%) compared to the control group. Mesalazine, EV 50, and EV 100 considerably boosted (*p* < 0.05) the HO-1 expression levels (100, 66.6, and 166.6%, respectively) compared to the AA group. (Figure 3B).

Figure 3C shows that AA persuaded a remarkable injury in the tight junction barrier expressed by a substantial decline (*p* < 0.05) in the colonic occludin gene expressions (130%) compared to the control group. Mesalazine, EV 50, and EV 100 considerably restored (*p* < 0.05) the occludin expression levels (100, 50, and 100%, respectively) compared to the AA group (Figure 3C).

### 2.9. Colon Interleukin-6 (IL-6) Level

The AA group exhibited a substantial rise (*p* < 0.05) in IL-6 level (508.86%) compared to the control group. Mesalazine treatment considerably diminished (*p* < 0.05) the colonic IL-6 level (54.89%) compared to the AA group. Furthermore, the EV 50 and EV 100 groups also showed a noteworthy decline in the IL-6 level (44.2 and 68.98%, respectively) compared to the AA group (Figure 3D).

### 2.10. Histopathological Examination

**Figure 4:**
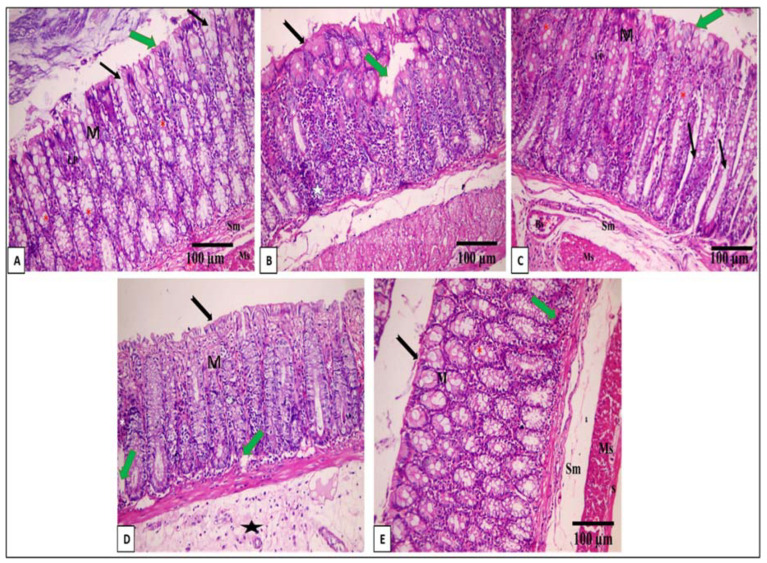
A photomicrograph of a section in the distal colon of an adult male albino rat: (**A**) control group I reveals mucosa (M), submucosa (Sm), and musculosa (Ms). Lamina propria (LP) of the mucosa contains closely packed crypts of Lieberkuhn (black arrow). The mucosa is lined by the surface epithelium with a brush border (green arrow) and crypts with abundant goblet cells (*) (H & E × 200, scale bar = 100 μm). (**B**) Sections in the distal colon of an adult male albino rat of the AA group showing focal loss of surface epithelium and part of the lamina propria (green arrow) detachment of the surface epithelium in some areas (black arrow) in addition to dense inflammatory cellular infiltration (star) (H & E × 200, scale bar = 100 μm). (**C**) Mesalazine group reveals normal histological structure of the colon as control group I: (H & E × 200, scale bar = 100 μm). (**D**) The EV 50 group shows apparently normal mucosa (M), intact surface epithelium (black arrow), and focal inflammatory cellular infiltrations with bleeding in lamina propria. Moreover, there are crypt cysts (green arrow), a mildly decreased number of goblet glands, and an increase in the thickness of the submucosa (star) (H & E × 200, scale bar = 100 μm). (**E**) The EV 100 group reveals the regaining of the normal appearance of the colon in the form of mucosa (M), submucosa (Sm), and musculosa (Ms). Lamina propria (LP) of the mucosa with few inflammatory cellular infiltrations (green arrow). The mucosa is lined by the surface epithelium, which has a brush border (black arrow), and crypts have abundant goblet cells (*) (H & E × 200, scale bar = 100 μm).

The histological features of the colon of the tested groups are exposed in Figure 4.

### 2.11. Immunohistochemical Features of Caspase-3, NF-kB, TNF-α, and COX-2

**Figure 5:**
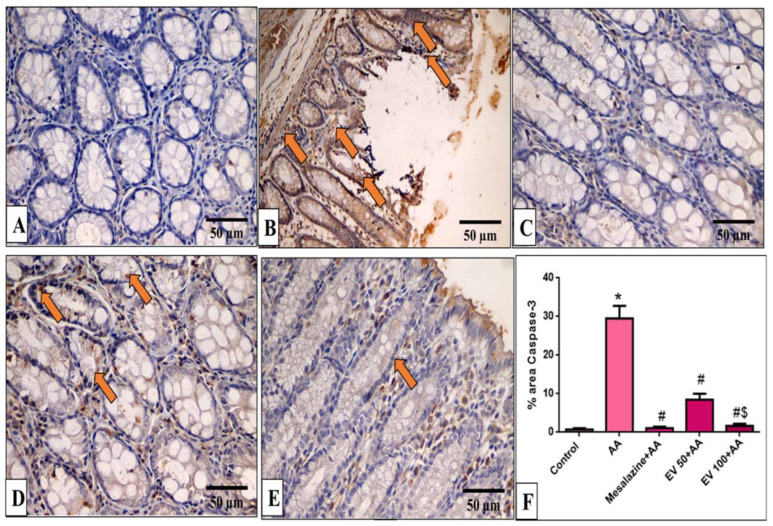
A photomicrograph of sections in the distal colon of adult male albino rats of various studied groups stained by caspase-3. (**A**) The normal control group shows a colonic gland with no caspase-3 immune reaction in all cells. (**B**) The AA group shows a strong positive caspase-3 immune reaction in epithelial and glandular cells (arrows). (**C**) The mesalazine group shows no caspase-3 immune reaction in all cells. (**D**) The EV 50 group IV moderate caspase-3 immune reaction in glandular cells (arrows). (**E**) The EV 100 group shows no caspase-3 immune reaction in the majority of cells and a weak immune reaction in a few surface epithelial cells (caspase-3 immune reaction in cells immunostaining × 400, scale bar = 50 μm). (**F**) Percent area of caspase-3-positive cells/1000 cells. Significant difference vs. * respective control, ^#^ respective AA group, ^$^ respective EV 50 group, each at *p* < 0.05.

**Figure 6:**
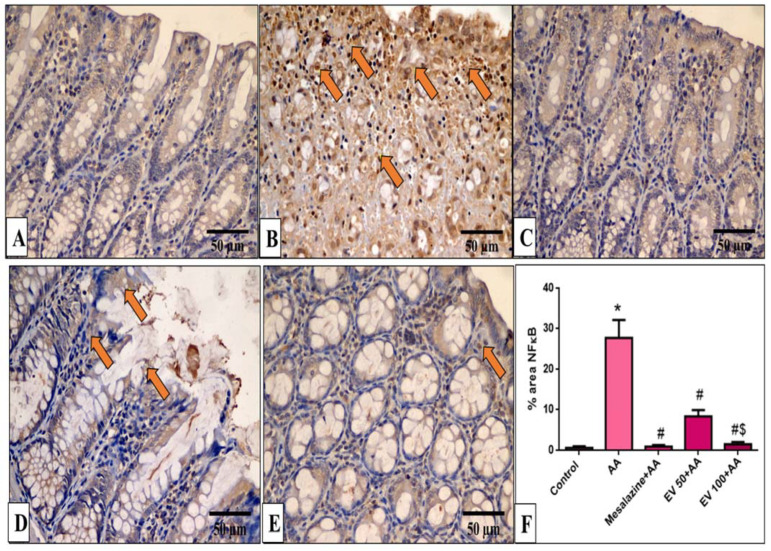
A photomicrograph of sections in the distal colon of adult male albino rats of various studied groups stained by NF-κB. (**A**) The normal control group shows a colonic gland with no NF-κB immune reaction in all cells. (**B**) The AA group shows a strong positive NF-κB immune reaction in surface epithelial and glandular cells (arrows). (**C**) The mesalazine group shows no NF-κB immune reaction in all cells. (**D**) The EV 100 group shows a moderate NF-κB immune reaction in surface epithelial cells (arrows). (**E**) The EV 100 group shows no NF-κB immune reaction in the majority of cells and a weak immune reaction in a few cells. (NF-κB immune reaction in cells immunostaining × 400, scale bar = 50 μm). (**F**) Percent area of NF-κB-positive cells/1000 cells. Significant difference vs. * respective control, ^#^ respective AA group, ^$^ respective EV 50 group, each at *p* < 0.05.

**Figure 7:**
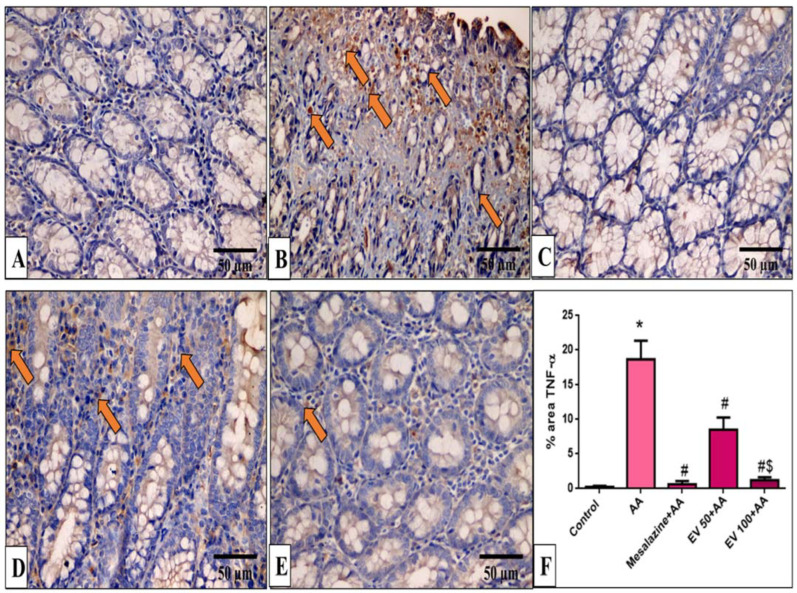
A photomicrograph of sections in the distal colon of adult male albino rats of various studied groups stained by TNF-α. (**A**) The normal control group shows a colonic gland with no TNF-α immune reaction in all cells. (**B**) The AA group shows a strong positive TNF-α immune reaction in surface epithelial and glandular cells (arrows). (**C**) The mesalazine group shows no TNF-α immune reaction in all cells. (**D**) The EV 50 group shows a moderate TNF-α immune reaction in some cells (arrows). (**E**) The EV 100 group shows no TNF-α immune reaction in the majority of cells and a weak immune reaction in a few cells (TNF-α immune reaction in cells immunostaining × 400, scale bar = 50 μm). (**F**) Percent area of TNF-α-positive cells/1000 cells. Significant difference vs. * respective control, ^#^ respective AA group, ^$^ respective EV 50 group, each at *p* < 0.05.

**Figure 8:**
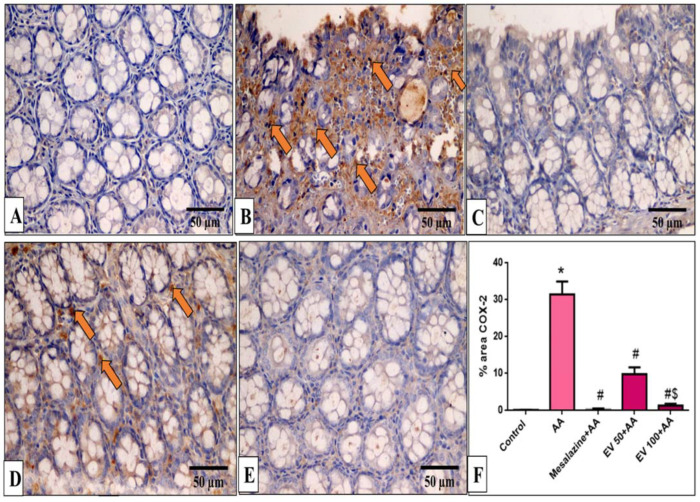
A photomicrograph of sections in the distal colon of adult male albino rats of various studied groups stained by COX-2 immunostaining. (**A**) The normal control group shows no COX-2 immune reaction in all cells. (**B**) The AA group shows a strong positive COX-2 immune reaction in surface epithelial and glandular cells (arrows). (**C**) The mesalazine group shows no COX-2 immune reaction in all cells. (**D**) The EV 50 group shows a moderate immune reaction in some glandular cells (arrows). (**E**) The EV 100 group shows no COX-2 immune reaction in all cells (COX-2 immunostaining × 400, scale bar = 50 μm). (**F**) Percent area of COX-2-positive cells/1000 cells. Significant difference vs. * respective control, ^#^ respective AA group, ^$^ respective EV 50 group, each at *p* < 0.05.

Caspase-3, NF-kB, TNF-α, and COX-2 immunohistochemical features of the colon of the different experimental groups are shown in Figure 5, Figure 6, Figure 7 and Figure 8.

Results of immune-staining quantification revealed that the AA group showed strong Capase-3 immunostaining (41.04-fold increase). EV 50- and EV 100-treated groups substantially reduced caspase-3 immunostaining by 71.39% and 94.3%, respectively, relative to the AA group (Figure 5F, *p* < 0.05).

In Figure 6F, the AA group significantly increased NF-κB immunostaining (41.3-fold increase). EV 50 and EV 100 treatments significantly decreased NF-ĸB immunostaining by 69.9% and 94.34%, respectively, with a more pronounced effect in the EV 100 group (Figure 6F, *p* < 0.05).

Also, as displayed in Figure 7F, the AA group induced a pronounced increase in TNF-α staining (71.89-fold increase). EV 50 and EV 100 treatment showed a significant reduction in TNF-α immunostaining by 54.44% and 93.5% compared to AA (Figure 7F, *p* < 0.05).

Results indicated that the AA group induced a marked increase in COX-2 staining (232-fold increase). EV 50 and EV 100 pre-treated groups significantly suppressed COX-2 immunostaining by 68.86% and 95.66% compared to AA (Figure 8F, *p* < 0.05).

## 3. Discussion

UC is a repeated colon inflammation characterized by the infiltration of inflammatory cells and expression of NF-kB-dependent pro-inflammatory biomarkers like TNF-*α* and IL-6 [19,20]. Also, ROS are released in this inflammatory disease. In addition, the mucosal integrity of the colon is usually lost [14,21]. Despite the great efforts that are currently being made to cure UC, it is still a poorly treatable ailment. This is in addition to the high risk of its progression to colon cancer. Thus, it is important to find novel therapies to obtain better clinical results.

Here, the AA-induced UC model was utilized to reveal the potential consequences of EV on colitis. Remarkably, there was a substantial reduction (*p* < 0.05) in the colon length and a substantial rise (*p* < 0.05) in the colon weight and colon weight/length ratio.

There was a vast release of ROS in the colon tissue after AA administration, and this is attributed to the destruction of the mucosa. This is demonstrated by the considerable reduction of the antioxidant enzymes (CAT and GSH-PX). EV extract-treated groups significantly replenished the capacity of antioxidant enzymes and alleviated oxidative stress, and this is consistent with a previous report [22].

According to the reported IBD pathophysiology, TLRs mediate innate protection in the bowel. Inappropriate levels of TLRs result in stimulation of pro-apoptotic signaling pathways and chronic inflammation [23,24]. Activation of TLR4 causes a rise in NF-kB expression (TLR4/NF-kB pathway) and, as a result, a vast release of inflammatory cytokines and enzymes like IL-1, IL-6, TNF-α, and COX-2 usually occurs, which have a major role in IBD [25,26]. So, it has been proposed that the TLR4/NF-ĸB pathway could be an objective for the treatment of IBD. Here, we found that AA-induced colitis has led to a rise in the expression of TLR4 by qRT-PCR and NF-kB immunostaining, which is in line with previous studies [27,28,29].

The EV 100-treated group displayed a pronounced decrease in TLR-4 expression and NF-kB immunostaining. HPLC of EV identified 18 compounds belonging to flavonoids and polyphenolic subclasses. This investigation revealed that EVME possesses antimicrobial and anti-inflammatory activities, which are likely due to the presence of several active constituents, such as phenolics, flavonoids, and glycoside derivatives. Naringenin is the most abundant flavonoid metabolite, followed by daidzein and quercetin [22,30].

Naringenin was reported to significantly improve colitis in dextran sulfate sodium (DSS)-induced UC in mice by impeding NF-κB and TLR4 protein activity [31,32]. Also, the expressions of iNOS, COX-2, TNF-α, and IL-6 were downregulated [33,34]. Since naringenin is the major compound in EV extract, this may explain its inhibitory effects on the TLR-4/NF-kB axis and ameliorating impact on AA-induced colitis. It has been revealed that flavonoids, as well as polyphenols, enhanced the therapeutic outcomes in the experimental colitis via decreasing colonic injury and inflammation [27]. All such reports established the anti-inflammatory potential of EV compounds and strengthened their use as an anti-inflammatory candidate in AA-induced UC [34].

In accordance with the preceding investigations, this study revealed increased levels of TNF-α immunostaining after AA-colitis induction and augmented levels of IL-6 [23]. TNF-α, in turn, recruits neutrophils toward the tissues. The MPO level was measured to indicate neutrophil accumulation [35,36]. It was noticed that there was a noteworthy rise in MPO activity after UC induction. Interestingly, the MPO activity was considerably declined by EV, which was demonstrated for the first time and may be partially explained by its constituents, which exhibit marked anti-inflammatory properties and a reduction in TNF-α levels in the colon [22,37]. Additionally, mesalazine resulted in a noteworthy decrease in MPO activity. Such findings are in line with previous studies [38].

As a result of the activity of COX-2, both prostaglandin E2 (PGE2) and thromboxane B2 are produced, which, in turn, induce intestinal hyperemia and edema [39]. Thus, activation of COX-2 could lead to an increase in ROS/RNS and a depletion of the antioxidative system. All of these consequences could lead to the induction of apoptotic injuries in the epithelium of the colon [40,41]. Here, AA-induced UC demonstrates an increase in COX-2 immunostaining. EV treatment significantly decreased the COX-2 immunostaining, and this could be due to inhibition of the ROS/RNS and reestablishment of the antioxidative activity. Our phytochemical investigation was in line with other previous research [37,42,43,44]. All of these phytochemicals revealed pronounced antioxidant and anti-inflammatory potentials, which might explain the beneficial impacts of EV extract.

Previous investigations found that apoptosis is associated with the pathophysiology of IBD [45]. The inflammatory response affects the function of the mucosal barrier and the intestinal integrity, which often leads to apoptosis [46,47,48]. In the current study, caspase-3 immunostaining was expressively expressed. Also, the expression of the tight junction protein, occludin, was reduced in the colon tissues of the AA-induced UC group. EV 100 treatment diminished caspase-3 immunostaining and upregulated occludin gene expression. Thus, the apoptotic injuries were prevented, and the intestinal integrity was maintained, which preserved the functions of the mucosal barriers. Thus, EV produced significant antiapoptotic impacts by diminishing the discharge of ROS and NO.

HO-1 is an antioxidant enzyme present at low levels in different normal tissues. Exposure to oxidants interrupts the transcription of antioxidant proteins like HO-1 and CAT [49,50,51]. HO-1 has immunomodulatory, antiproliferative, anti-inflammatory, and antiapoptotic effects [52]. Therefore, in our study, HO-1 expression levels were markedly suppressed in the AA-induced UC. EV-treated groups significantly replenished HO-1 expression levels, confirming EV extract’s anti-inflammatory, antioxidant, and antiapoptotic properties. These effects may be due to their flavonoids, such as naringenin, daidzein, kaempferol, and quercetin derivatives, as well as phenolic compounds, such as chlorogenic acid and cinnamic acid. All these bioactive molecules were previously reported for their anti-inflammatory, antioxidant, and antiapoptotic impacts [37,53,54,55].

EV ameliorated the morphological mucosal damage recorded by the histological studies, which is consistent with other studies [23,56]. EV lessened the detrimental impact of AA by refining all the studied factors. Our study is the first investigation indicating the possible ameliorative properties of EV on AA-induced UC in rats. All these effects might prove the plausible protective action of *Encephalartos villosus* on AA-induced UC, which was first demonstrated in this study.

## 4. Materials and Methods

### 4.1. Animals

Fifty male Wistar albino rats with weights ranging from 150 to 170 g were obtained from Cairo University (Egypt). Animals were preserved in standard conditions. The experimental techniques were accepted by the research ethical committee (Faculty of Pharmacy, Tanta University, Egypt) (TP/RE/6/23 p-0031).

### 4.2. Plants, Drugs, and Chemicals

Mesalazine was obtained from EL-Pharonia pharmaceutical company (Cairo, Egypt). All other chemicals were gained from Sigma Aldrich (St. Louis, MO, USA).

#### 4.2.1. *Encephalartos villosus* Extract Preparation

*Encephalartos villosus* Lem. leaves were collected from Al Orman Botanical Garden on 15 July 2021. Dr. Esraa Ammar, Plant Ecology, Tanta University, recognized the plant. A voucher sample (PG-G-106-E) was deposited at the Herbarium of the Faculty of Science, Tanta University. The plant was dried at room temperature for ten days, then in an oven at 40 °C for two days, and then ground. The powder (300 g) was extracted by methanol (four L × three times) at two-day intervals using the cold maceration method at room temperature to yield 31.39 g of EV extract [22].

#### 4.2.2. High-Performance Liquid Chromatography (HPLC) Analysis

HPLC analysis of the EV is performed following [57] with some modifications. The SCRE was analyzed using an Agilent 1260 series instrument and Eclipse C18 column (4.6 mm × 250 mm i.d., 5 µm). Separation was performed at a flow rate of 0.9 mL/min. The mobile phase consisted of water (reservoir A) and 0.5% trifluoroacetic acid in acetonitrile (reservoir B) at a concentration of 0.1%. The mobile phase was sequentially programmed with a linear gradient as follows: 0 min (82%A); 0–5 min (80%A); 5–8 min (60%A); 8–12 min (60%A); 12–15 min (82%A); 15–16 min (82%A); and 16–20 (82%A). A multi-wavelength UV detector was used for detection at 280 nm. The injection volume for each sample solution was 5 μL. The column temperature was retained at 40 °C.

### 4.3. Experimental Procedure

#### 4.3.1. Induction of UC

It was provoked on the 15th day by the intra-rectal delivery of 2 mL of 3% AA via a rubber catheter into rats’ rectum under anesthesia. Then, animals were left in a vertical position for one minute after administration to prevent the leakage of AA [58].

#### 4.3.2. Design

Fifty animals were grouped into five categories (*n* = 10). Group I (control group) administered 2 mL of saline intra-rectally. The remaining groups administered AA intra-rectally and were subdivided into group II (untreated AA group), group III (mesalazine group) receiving 100 mg/kg of mesalazine [59] orally daily for 14 days, group IV (EV 50 group) receiving 50 mg/kg of EV orally daily for 14 days, and group V (EV 100 group) receiving 100 mg/kg of EV orally daily for 14 days [22]. After the last dose, rats fasted for 12 h before intra-rectal delivery of either AA or saline.

#### 4.3.3. Tissue Collection

Rats were anesthetized, and then blood was collected via cardiac puncture. Blood was then centrifuged and utilized for further investigations. Rats were then euthanized, and colons were isolated, washed, and dried using filter papers. Colons were then weighed and stretched to detect the distance between the colorectal junction and the distal rectum end [60]. The colon weight was divided by its length (gm/cm) to calculate the colon weight/length ratio [61].

The colons were allocated into two sectors. One section was utilized for histopathological and immunohistochemistry studies. The other section was used for the biochemical examination.

### 4.4. Colorimetric Determination of Colon Catalase and Glutathione Peroxidase

CAT and GSH-Px activities were evaluated in the homogenate of colon tissues using CAT and GSH-Px assay kits Cayman (Ann arbor, MI, USA) and Elabscience (Houston, TX, USA), respectively, according to the instructions of the manufacturers.

### 4.5. Colorimetric Determination of the Colon Myeloperoxidase Activity

MPO is an indicator of neutrophil infiltration. Thus, it was assessed in the colon tissue homogenate using an MPO assay kit (Abcam, Cambridge, UK).

### 4.6. ELISA for Interlukin-6 Levels

The IL-6 level was evaluated in the colon tissue homogenates by an ELISA kit (Sun Red Biotechnology Co., Shanghai, China). The intensity of the color was detected at 450 nm by an ELISA reader (Labnics, Glasgow, UK).

### 4.7. Quantitative Real-Time (qRT-PCR) for TLR-4, Heme Oxygenase-1, and Occludin

The extracted total RNA using TRIzols (Life Technologies, Carlsbad, CA, USA) [62] was reverse-transcribed into cDNA using the QuantiTects reverse transcription kit (Qiagen, Hilden, Germany). The cDNA was amplified using the Maximas SYBR green/fluorescein qPCR master mix using the primers listed in Table S1.

### 4.8. Histopathology of Colon Sections

Colons were fixed in formalin, and they were embedded in paraffin wax, sliced into very thin sections, and stained by hematoxylin and eosin (H&E) [37]. The stained sections were visualized by a light microscope.

### 4.9. Immunohistochemical Detection of Caspase-3, NF-kB, TNF-α, and COX-2

The immunohistochemical investigations were carried out using the monoclonal antibodies of caspase-3, NF-kB, TNF-α, and COX-2 (Santa Cruz Biotechnology, Dallas, TX, USA), as previously reported [63,64,65]. Immuno-stained slides were image-analyzed using ImageJ software.

### 4.10. Statistics

Results are revealed as the mean ± standard error of the mean (SEM). An ANOVA test was utilized to investigate the difference between groups at *p* < 0.05 using Prism version 6 (San Diego, CA, USA).

## 5. Conclusions

This investigation revealed that EV successfully replenished the damage in the colonic mucosa via the administration of AA. It exerted potent anti-inflammatory, antioxidant, and antiapoptotic impacts. The studied molecular mechanisms for such effects could be due to suppressing the TLR4/NF-kB signaling pathway owing to its active constituents. Additional studies have to be conducted to endorse the clinical use of *Encephalartos villosus* in ulcerative colitis patients.

## Figures and Tables

**Table 1:** Chemical composition analysis of the phenolic and flavonoid compounds in *Encephalartos villosus* by HPLC.

Identified Compound	RT (Min)	Area %	Area [mAU*s]	Conc. (µg/g)
Gallic acid	3.384	3.4543	345.99289	1008.68
Chlorogenic acid	4.228	14.5071	1453.08276	7595.27
Catechin	4.460	1.8801	188.31728	1883.93
Methyl-gallate	5.630	1.4192	142.15103	333.51
Caffeic acid	6.053	3.3520	335.75253	926.40
Syringic acid	6.615	0.0768	7.68790	22.68
Pyrocatechol	7.091	0.0551	5.51670	28.18
Rutin	8.024	0.3438	34.43813	91.14
Ellagic acid	8.515	0.4069	40.75592	508.97
Coumaric acid	9.130	1.3329	133.50352	157.58
Vanillin	9.711	0.5434	54.42558	74.84
Ferulic acid	10.065	2.5549	255.90369	580.48
Naringenin	10.492	34.7159	3477.27783	11,476.89
Daidzein	12.208	13.0087	1302.99866	3239.40
Quercetin	12.450	12.3932	1241.34583	5485.34
Cinnamic acid	13.336	9.1824	919.74677	702.02
Apigenin	14.496	ND	ND	ND
Kaempferol	14.832	0.4681	46.88348	179.35
Hesperetin	16.147	0.3054	30.59113	64.45

ND: Not detected.

**Table 2:** Influence of EV treatment on colon weight, colon length, and colon weight/length ratio in AA-induced UC in rats.

	Colon Weight (gm)	Colon Length (cm)	Colon Weight/Length Ratio (gm/cm)
Control	1.136 ± 0.092	12.9 ± 0.42	8.8 ± 0.57
AA	1.418 ± 0.143 *	10.1 ± 0.74 *	14.14 ± 2.27 *
Mesalazine + AA	1.064 ± 0.1 ^#^	12 ± 0.8 ^#^	8.89 ± 1.03 ^#^
EV 50 + AA	1.23 ± 0.08	10.6 ± 0.65	11.61 ± 0.93 ^#^
EV 100 + AA	1.122 ± 0.098 ^#^	12.58 ± 0.94 ^#$^	8.98 ± 0.6 ^#$^

Substantial difference vs. * control, ^#^ AA group, and ^$^ EV 50 group, each at *p* < 0.05.

## Data Availability

All data is contained within the article and Supplementary Material.

## Supplementary Materials

The following supporting information can be downloaded at: https://www.mdpi.com/article/10.3390/ph16101431/s1, Table S1: Primers used and their sequences. Figure S1: Chromatogram for the standard flavonoids and phenolic compounds.
