# Supplementary material for: Plausible Protective Role of Encephalartos villosus Extract in Acetic-Acid-Induced Ulcerative Colitis in Rats
Source: Pharmaceuticals (Basel). 2023 Oct 9;16(10):1431. doi: 10.3390/ph16101431 (PMC10609761; doi:10.3390/ph16101431)
Supplement: Supplementary file 1 [file pharmaceuticals-16-01431-s001.zip › pharmaceuticals-2614490-supplementary.pdf]

pharmaceuticals

MDPI

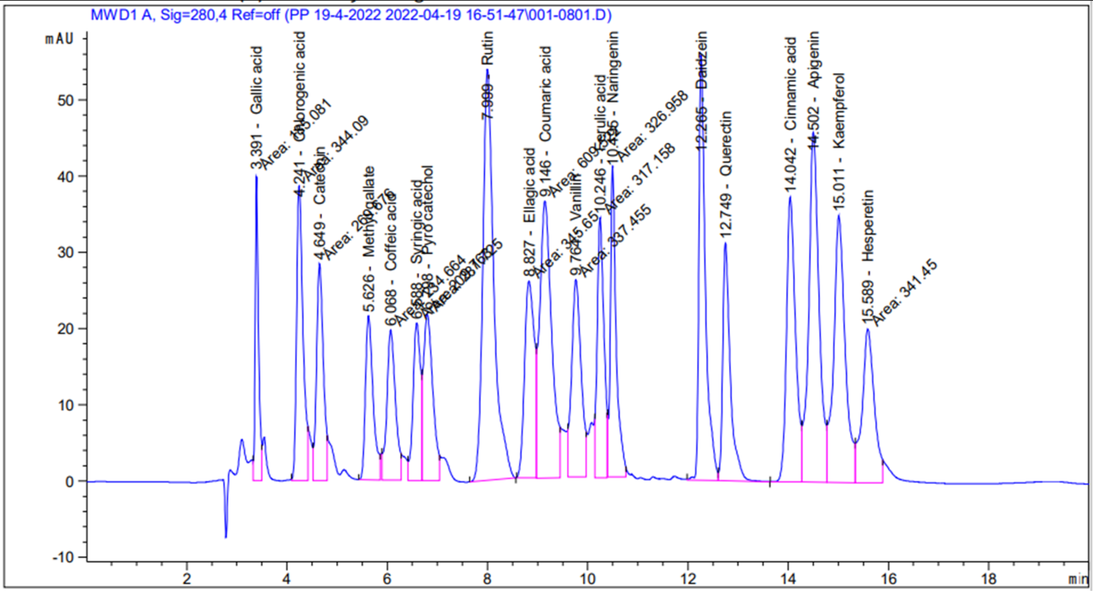

Figure S1: Chromatogram for the standard flavonoids and phenolic compounds

Table S1. Primers used and their sequences.

**Genes**	**Sequences (5'-3')**
**β-actin**	AGGCGTCCTTCCTTATATGCTA<br>GGCTGTATTCCCCTCCATCG
**HO-1**	AGGTACACATCCAAGCCGAGA<br>ATCACCAGCTTAAAGCCTTCT
**TLR-4**	AGACATCCAAAGGAATACTGCAA<br>GCCTTCATGTCTATAGGTGATGC
**Occludin**	ACAAAGAGCTCTCTCGTCTCG<br>CATAGTCTCCCACCATCCTC
